# Peptidomic Analysis of Neonate Umbilical Cord Blood for the Identification of Endogenous Peptides Involved in Hypoxic–Ischemic Encephalopathy

**DOI:** 10.3389/fped.2021.718704

**Published:** 2021-08-25

**Authors:** Xiaohua Dong, Jing Zhao, Yinyin Shen, Qing Sun, Xiaohui Wu, Yanqing Zhu, Lingling Yu, Yingmin Zhao

**Affiliations:** ^1^Tongren Hospital, Shanghai Jiao Tong University School of Medicine, Shanghai, China; ^2^Department of Pediatric, Jingjiang People's Hospital Affiliated to Yangzhou University, Jingjiang, China

**Keywords:** hypoxic-ischemic encephalopathy, neonates, peptides, peptidomic, KEGG

## Abstract

Neonatal hypoxic–ischemic encephalopathy (HIE) is a common neurological disorder triggered by perinatal cerebral ischemia and hypoxia. Accumulating evidence has shown that peptides have neuroprotective effects in nerve injury. However, the function of endogenous peptides in the pathogenesis of HIE has not been studied. In the present study, a comparative peptidomic profile was performed in the serum of the human umbilical cord blood with HIE (three patients) and the control group (three health control) by liquid chromatography–mass spectrometry (LC-MS). Our study demonstrated that a total of 49 peptides derived from 25 precursor proteins were differentially expressed in the serum of HIE compared with normal controls, including 33 upregulated peptides and 16 downregulated peptides. Each of the differentially expressed peptides has specific characteristics, including pI, Mw, and cleavage pattern. Gene ontology (GO) and Kyoto Encyclopedia of Genes and Genomes (KEGG) analyses indicated that the precursor proteins of differentially expressed peptides participate in the different biological process. Moreover, among the 49 differentially expressed peptides, 21 peptides were identified from the fibrinogen chain family, which plays a role in neurological diseases, suggesting that these peptides may play an important role in maintaining brain health. In conclusion, our results showed a comparative peptidomic profile from human umbilical cord blood of HIE patients and normal controls. These dysregulated peptides may have potentially important functions in umbilical cord blood with HIE and may be involved in the pathogenesis of the HIE.

## Introduction

Neonatal hypoxic–ischemic encephalopathy (HIE) is the major cause of morbidity and mortality in neonates, mainly because less oxygen and glucose are delivered to the brain (1, 2). Asphyxia is the most common pathway leading to HIE, and it can occur before, during, or after delivery due to complicating factors (3, 4). In high-income countries, the incidence rate of HIE in neonates is 0.8 to 2 per thousand. The prognosis of HIE is closely related to the clinical grade of encephalopathy (5). Approximately 50% of moderate HIE is associated with long-term nerve injury, including death, epilepsy, neurodevelopmental disability, and intellectual disability (6).

The cellular pathophysiology of hypoxic–ischemic brain injury is complicated and mainly initiates the apoptotic pathway caused by secondary energy failure (3, 7). Hypothermia therapy is considered to be the most effective method for the treatment of moderate and severe HIE by decreasing the brain's metabolic rate and reducing neurological sequelae without adverse effects (8–12). The majority of the studies have illustrated that therapeutic hypothermia must start within 6 h after birth to improve the neurological function of the survivors (13, 14). HIE is a rapidly developing encephalopathy, and the neurologic examination of infants will change rapidly over time. Routine clinical investigation and instrument examination have great risk for the early and accurate diagnosis of HIE (15). Therefore, early and accurate diagnosis of newborns with moderate and severe HIE is an urgent problem to be solved.

Peptidomics is the quantitative analysis of large numbers of low-molecular-weight polypeptides that are intermediate products of protein hydrolysis in living cells (16). Accumulated studies indicate that peptides are involved in various biological processes and are considered to be used in either diagnosis or further treatment (17–19). However, the peptide spectrum in cord blood serum of neonates with HIE has not been elucidated. In this study, we identified a total of 49 peptides that are differentially expressed in the serum of umbilical cord blood of neonates with HIE compared with healthy controls. These differentially expressed peptides can be used as rapid screening biomarkers for HIE and may be involved in the pathogenesis of HIE.

## Materials and Methods

### Sample Collection

Serum samples were collected from the umbilical cord blood of the neonates at the Jingjiang People's Hospital affiliated to Yangzhou University from October 2019 to February 2020. The serum samples were divided into two groups according to clinical diagnosis: the HIE group (*n* = 3) and normal controls (*n* = 3). The clinical features of HIE and control samples are listed in Table 1. The diagnosis of HIE was confirmed by regular examination: an Apgar score of 2–6 for 1 min and <8 after first 5 min after birth was indicated as HIE. This study was approved by the Medical Ethics Committee of Jingjiang People's Hospital in China [approval no. (2020)38].

**Table 1:** Clinical features of HIE and control samples.

**Number**	**Mother Age/Year**	**Gestational week**	**Neonate Age/Day**	**Sex(M/F)**	**1 min Apgar**	**5 min Apgar**
NC-1	36	38+3	1	M	10	10
NC-2	30	38+5	1	M	10	10
NC-3	30	39	1	F	10	10
HIE-1	30	41	1	M	3	6
HIE-2	30	38	1	F	4	7
HIE-3	31	38+2	1	F	3	7

*M, male; F, female; NC, Negative Control; HIE, Hypoxic Ischemic Encephalopathy*.

### Peptide Extraction and Labeling

To a 300-μl serum sample, 100% TCA (trichloroacetic acid) was added to the final concentration of 20%, and then an equal volume of CHL3 (trichloromethane) was added. The sample was then mixed thoroughly and placed on ice for 1 h. The sample was then centrifuged for 10 min at 150 × g at 4°C, and to the supernatant 10 μl of water and 10 μl of methanol were added, after which the sample was mixed thoroughly and centrifuged for an additional 10 min at 150 × g at 4°C. To the supernatant, 1 M TEAB was added to four times the volume of the supernatant, adjusting the pH to 2–3.

Twenty milligrams of C18 column material was activated with 1 ml of methanol, vortexed, and centrifuged, and the supernatant was discarded. One milliliter of 0.1% FA (formic acid) was added for acidification, followed by vortexing and centrifugation, and the supernatant was discarded. The sample was eluted with 1 ml of 0.1% FA plus 3% can (acetonitrile) three times, and the supernatant was discarded. Then, 800 μl of ACN and 200 μl of 0.1% FA were added and mixed for 30 min, and the supernatant was taken. Peptides were quantitated with the BCA method and were then freeze-dried.

Equal amounts of peptide were dissolved in 0.5 M TEAB and labeled according to the instructions of the iTRAQ-8 kit (SCIEX, Framingham, MA, USA). The labeled peptides were mixed, and the peptide samples were separated using an Ultimate 30 HPLC system (Dionex, Thermo Scientific, Waltham, MA, USA). The column used was a C18 (Durashell, 5 μm, 100 Å, 4.6 × 250 mm). The peptides were separated by increasing the ACN concentration under alkaline conditions. The flow rate was 1 ml/min, and fractions were collected every minute. A total of 42 secondary fractions were collected and combined into 12 components. The combined components were desalted on a Strata-X column and vacuum-dried.

### Liquid Chromatography–Mass Spectrometry Analysis

A TripleTOF 560 and an MS system (SCIEX, USA) were used for MS data acquisition. The peptide samples were dissolved in 2% AC/0.1% FA, added to a C18 capture column (5 μm, 10 μm × 20 mm), and eluted on a C18 analytical column (3 μm, 75 μm × 150 mm) at 30 nl/min for 90 min. For information-dependent acquisition (IDA), the mass spectrometer (MS) was scanned with 250 ms of ion accumulation time, and the MS2 spectra of 30 precursor ions were collected with 50 ms of ion accumulation time. The MS1 spectra were collected in the range of 350–1,500 m/z, and the MS2 spectra were collected in the range of 100–1,500 m/z. The dynamic removal time of precursor ions was 15 s.

### Protein Identification

ProteinPilot (V4.5) software was used for protein identification (unused score ≥ 1.3, conf ≥ 95%) and quantification of iTRAQ-labeled proteomics. Peptides with a fold change ≥ 1.2 or ≤ 0.83 with a Student's test *p* ≤ 0.05 were selected as differentially expressed peptides.

### Bioinformatics

The ProParm tool (https://web.expasy.org/protparam/) was used to calculate the isoelectric point (pI) and Mw of each peptide. The GO and KEGG pathways were predicted using websites http://geneontology.org/ and http://www.genome.jp/kegg/ or http://www.kegg.jp/. The interaction network function of the identified peptide precursor proteins was analyzed by STRING (https://string-db.org, Version 11.0). PeptideRanker (http://distilldeep.ucd.ie/PeptideRanker/) was used to predict bioactive peptides (20).

### Statistical Analysis

Data were analyzed using the SPSS 20.0 software package with an independent-sample *t*-test for comparisons between two groups. A *p* < 0.05 was indicated as a statistically significant difference.

## Results

### Umbilical Cord Serum Peptidomic Analyses of Neonates With HIE

**Figure 1:**
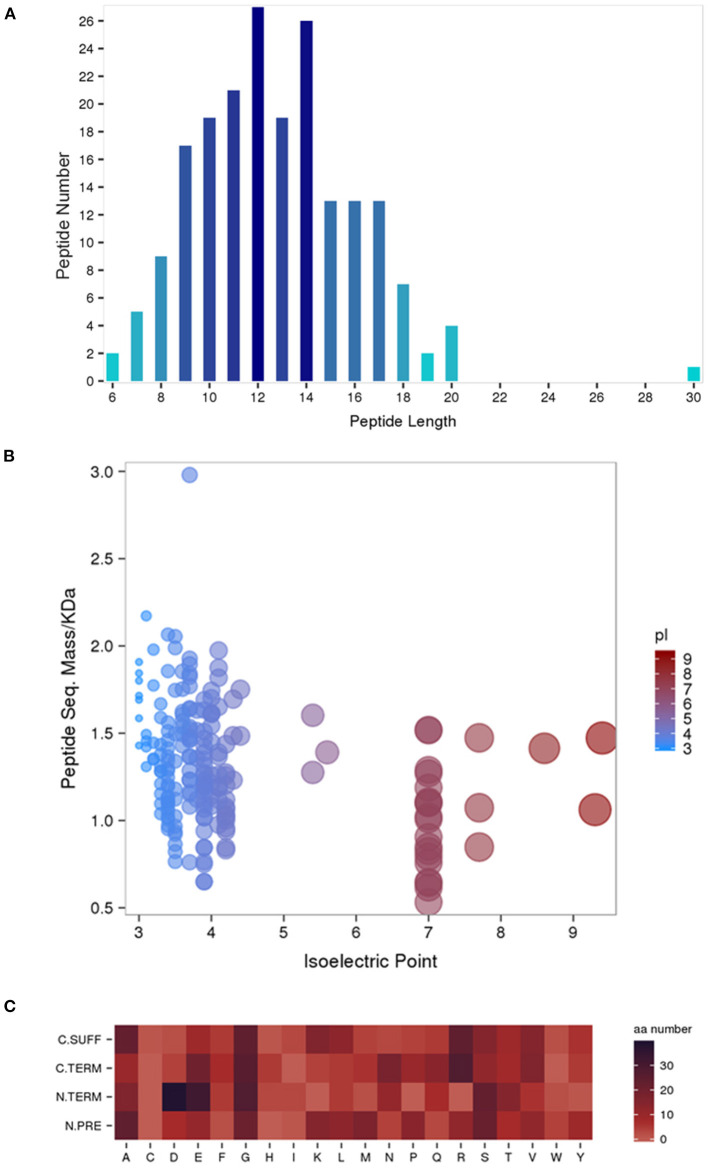
General properties of the identified serum peptides by iTRAQ. Peptide lengths **(A)**. The molecular weight (Mw) vs. isoelectric point (PI) distribution of the identified total peptide segments **(B)**. Heat map of the amino acid distribution of the cleavage sites of the precursor proteins **(C)**. N. PRE represents the former amino acid at the N-terminus of the peptide, N. TERM represents the amino acid at the N-terminus of the peptide, **(C)** TERM represents the amino acid at the C-terminus of the peptide, and C. SUFF represents the latter amino acid at the C-terminus of the peptide.

A total of 198 peptides derived from 62 precursor proteins were identified and quantified by performing iTRAQ in the serum of the human umbilical cord samples of the HIE newborns and normal-born babies. By analyzing the general properties of the identified serum peptides, we found that the lengths of most peptides ranged from 8 to 18 amino acids (Figure 1A). In addition, the relationship between the molecular weight (Mw) and the isoelectric point (PI) was also investigated, and the most distributed PI 3.0–PI 5.0 peptides had Mws between 1.0 and 2.0 kDa, whereas the most distributed PI 7.0–PI 9.0 peptides had Mws between 0.5 and 1.5 kDa (Figure 1B). Furthermore, the cleaved amino acids from the precursor proteins for the preceding peptides had a certain pattern (Figure 1C). Alanine (A) and glycine (G) were the high-frequency amino acid break sites of the protein precursor groups.

### Analysis of Differentially Expressed Peptides in the Samples

**Figure 2:**
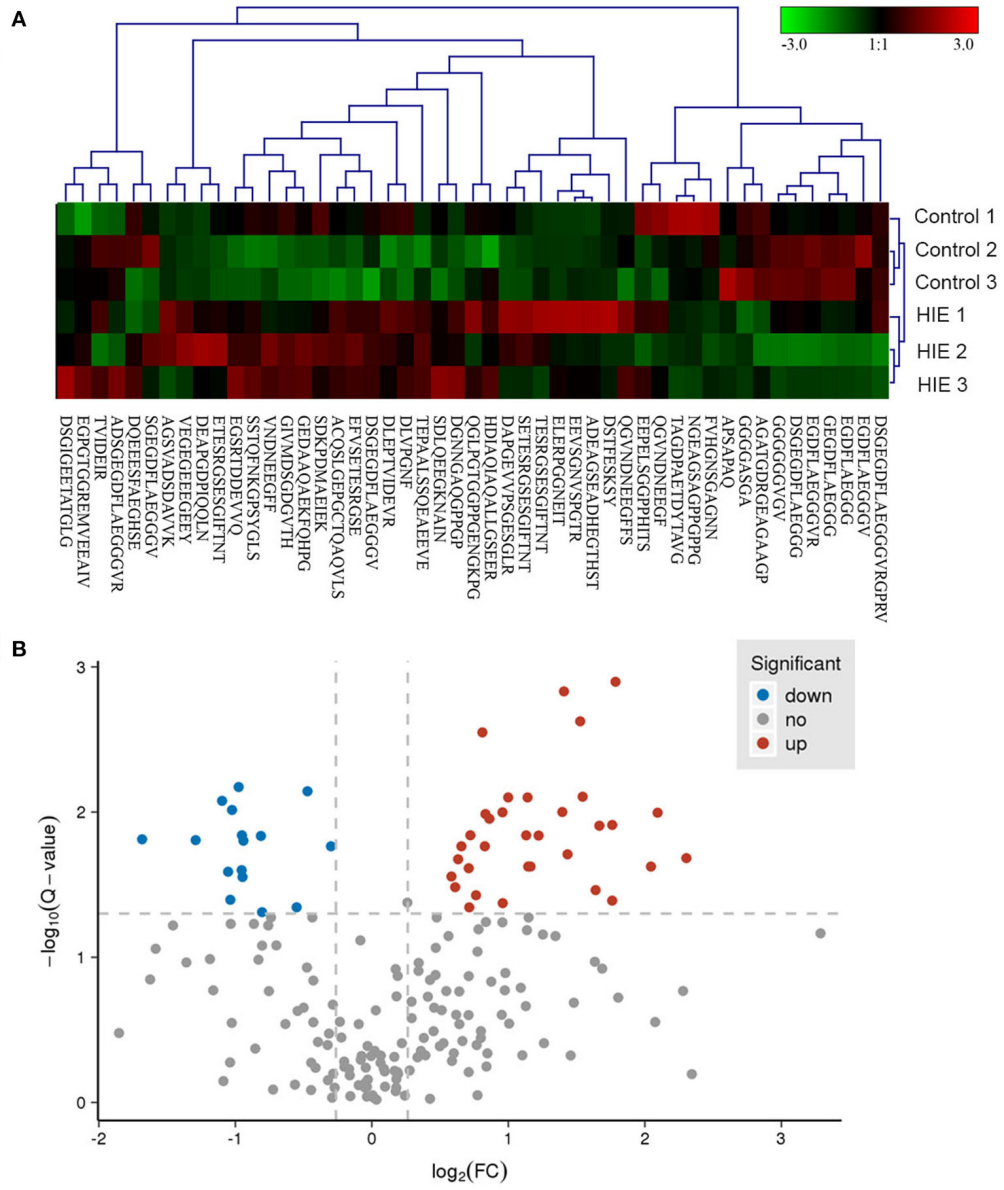
Hierarchical clustering and volcano map of differentially expressed peptides. Hierarchical clustering of differentially expressed peptides **(A)**. Volcano map of differentially expressed peptides **(B)**.

A subset of 49 peptides were found to be significantly differentially expressed (*p* < 0.05) in the HIE group compared with the normal group, representing 41.4% of the total identified peptidomes. These 49 peptides included 33 upregulated peptides and 16 downregulated peptides (FDR > 1.2 or FDR <0.8), which were derived from 25 precursor proteins. Hierarchical clustering (Figure 2A) and volcano mapping (Figure 2B) showed these differentially expressed peptides between the two groups of newborn HIE and normal-born babies. The sequences of some differentially expressed peptides in the HIE group compared with the control group are shown in Table 2.

**Table 2:** Part peptides of differentially expressed in HIE serum compared with control (Foldchange >2 or < 0.5).

**Sequence**	**Length**	**pI**	**Mw**	**Protein name**	**Fold-chage**	***P*-value**
DLVPGNF	7	3.7	1064.58	FGA	4.9385	0.020755
TEPAALSSQEAEEVE	15	3.3	1892.91	LAT	4.2675	0.01010
DEAPGDPIQQLN	12	3.4	1599.80	FERMT3	4.1257	0.023729
ELERPGGNEIT	11	4	1517.79	FGA	3.4465	0.001266
ETESRGSESGIFTNT	15	4	1917.92	FGA	3.3892	0.012264
DAPGEVVPSGESGLR	15	3.9	1772.92	TMEM40	3.3882	0.040725
GIVMDSGDGVTH	12	4.1	1506.72	ACTB	3.1751	0.012416
EEPELSGGPPHITS	14	4	1752.88	ESYT1	3.1151	0.034471
TESRGSESGIFTNT	14	4.3	1788.87	FGA	2.9164	0.007838
EFVSETESRGSE	12	3.8	1659.78	FGA	2.8806	0.00237
SETESRGSESGIFTNT	16	4	2004.95	FGA	2.7011	0.019539
DGNNGAQGPPGP	12	3.7	1399.66	COL1A2	2.6527	0.001475
EEVSGNVSPGTR	12	4.3	1534.78	FGA	2.6303	0.009987
AGSVADSDAVVK	12	4.1	1463.77	LGALSL	2.332	0.014510
DQEEESFAEGHSE	13	3.6	1796.76	CAVIN2	2.2385	0.023729
VEGEGEEEGEEY	12	3.2	1658.71	TUBA1A	2.212	0.023729
SDLQEEGKNAIN	12	3.9	1662.83	PARVB	2.2051	0.007929
EGPGTGGREMVEEAIV	16	3.8	1933.97	PLEKHG2	2.1894	0.014462
GEGDFLAEGGG	11	3.4	1311.62	FGA	0.4916	0.009668
FVHGNSGAGNN	11	7.7	1376.67	TUBB1	0.4874	0.040194
EGDFLAEGGGVR	12	3.9	1509.77	FGA	0.482	0.025770
GGGGASGA	8	7	836.424	INSM1	0.4674	0.008365
GGGGGGVGV	9	7	919.50	BHLHE22	0.4087	0.015600
TAGDPAETDYTAVG	14	3.4	1670.79	TLN1	0.3114	0.01539

### Features of Differentially Expressed Peptides

**Figure 3:**
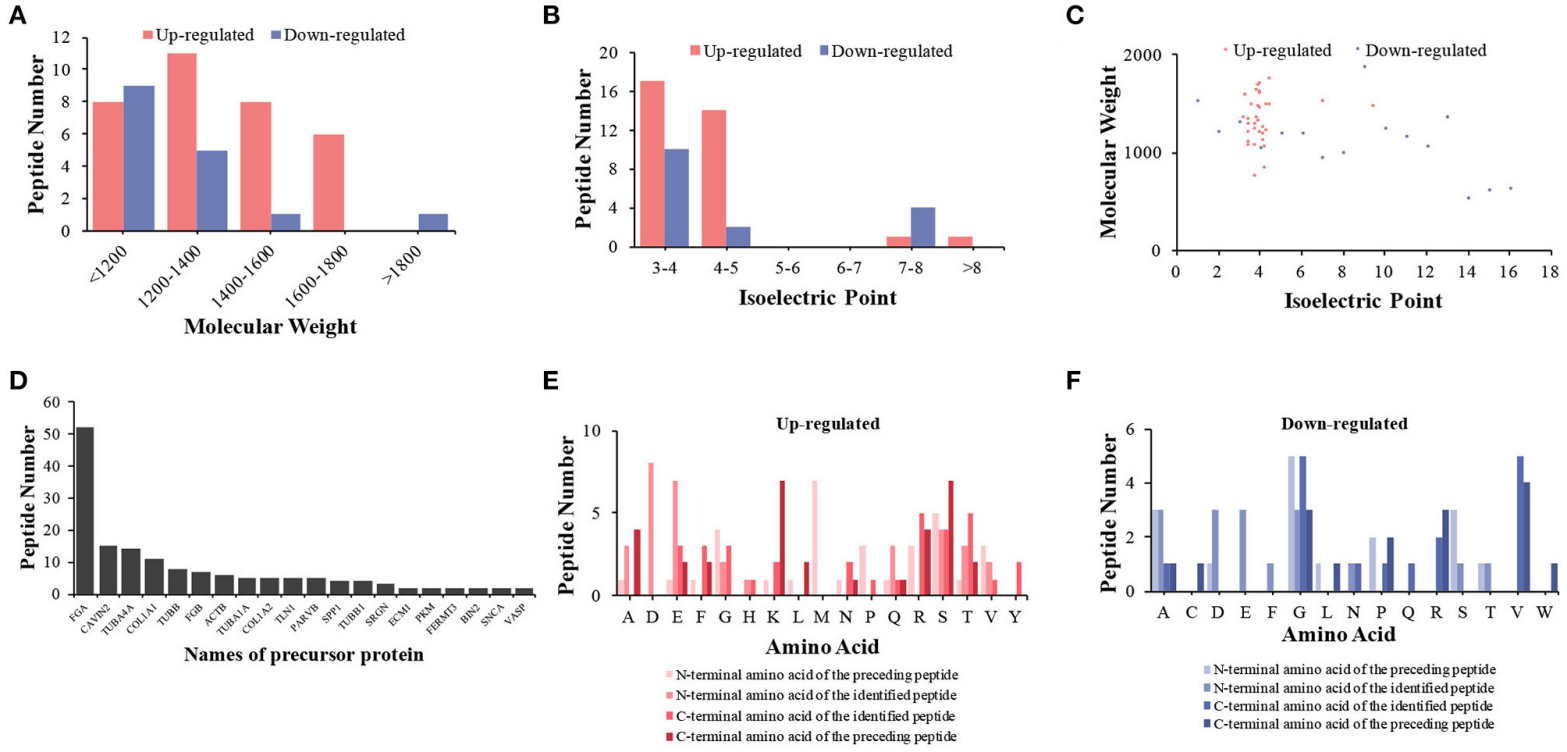
Features and cleavage patterns of differentially expressed peptides. Molecular weights of upregulated and downregulated peptides **(A)**. Isoelectric points of upregulated and downregulated peptides **(B)**. Scatter plot of isoelectric point vs. molecular weight in upregulated and downregulated peptides **(C)**. Peptide numbers for each protein precursor **(D)**. Distribution of the cleavage sites in upregulated peptides **(E)**. Distribution of the cleavage sites in downregulated peptides **(F)**.

We first analyzed the general characteristics of the differentially expressed peptides between HIE umbilical cord serum and relative control umbilical cord serum. The distribution of Mw, pI, and Mw vs. pI of the differentially expressed peptides was analyzed (Figures 3A–C). We found that the distribution of Mw, pI, and Mw vs. pI was in a large-scale range. As shown in Figure 3, the Mws of most differentially expressed peptides were ranged from 1,200 to 1,800 D, whereas the pIs were mostly distributed in the ranges of 3–5 and 7–8.

### Analysis of Differentially Expressed Peptide Cleavage Patterns

Peptides are generated by the protein degradation or restricted proteolysis from their precursor protein (21). According to the inconsistency of peptide breakage sites, these precursor proteins can produce multiple peptides. We found 20 precursor proteins containing more than one peptide in our samples (Figure 3D). We further investigated the cleavage patterns of all differentially expressed peptides to determine the possible functional changes of those peptides. The results showed that among the upregulated peptides, the commonly used cleavage sites for the preceding peptide of the N-terminal amino acid of the preceding peptide, the N-terminal amino acid of the identified peptide, the C-terminal amino acid of the preceding peptide, and the C-terminal amino acid of the identified peptide were methionine (M), lysine (K) or serine (S), aspartic acid (D), and arginine (R) or threonine (T), respectively (Figure 3E). Among the downregulated peptides, the most frequent cleavage sites were glycine (G), alanine (A)/aspartic acid (D)/glutamic acid (E) or glycine (G), glycine (G) or valine (V), and valine (V) (Figure 3F). The bioactivity of peptides could be predicted with the cleavage sites and sequences of the peptides.

### Gene Ontology and Pathway Analysis

**Figure 4:**
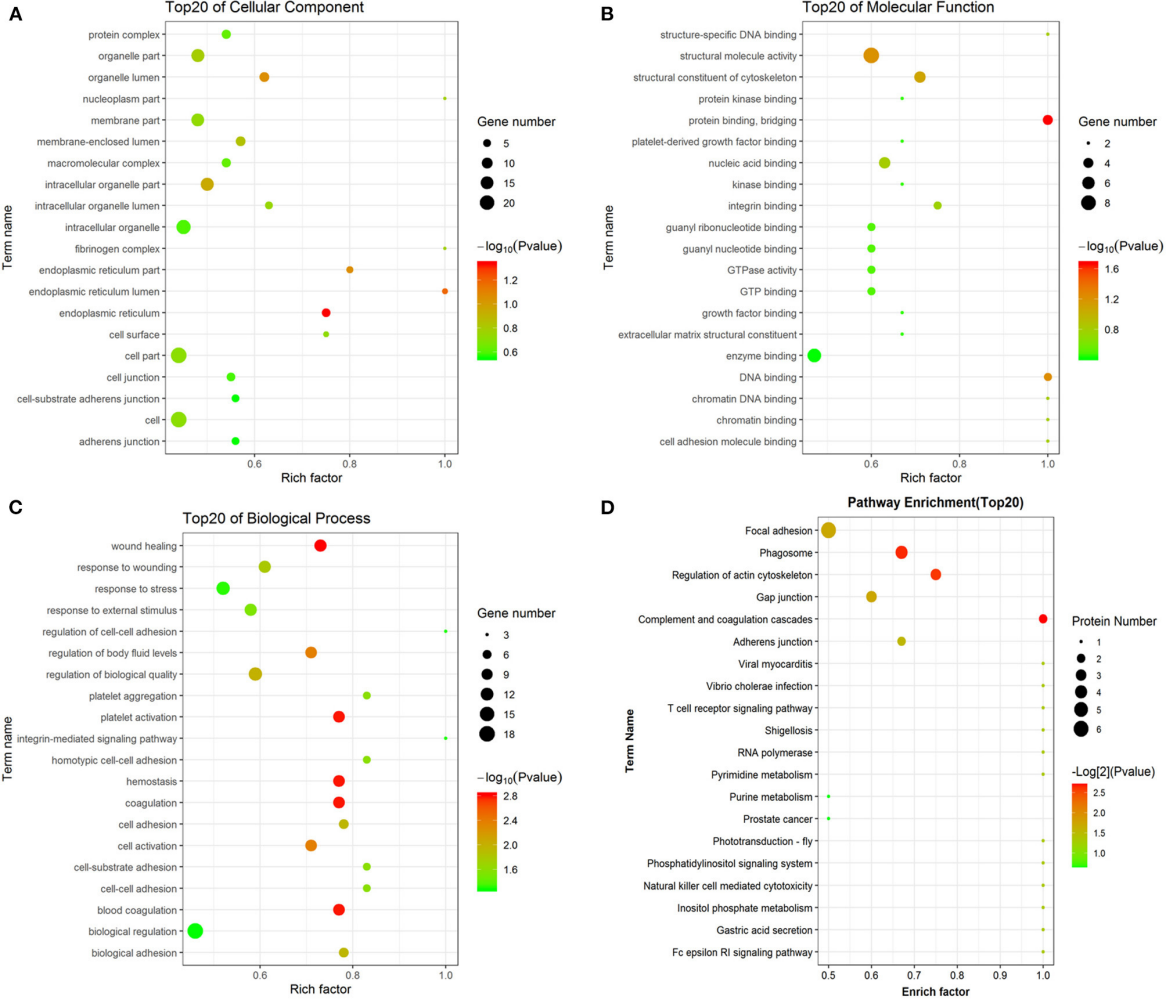
GO and pathway analyses of precursor proteins of differentially expressed peptides. The cellular component categories **(A)**. The molecular function categories **(B)**. The biological process categories **(C)**. Pathway analysis **(D)**.

To determine the potential function of the differentially expressed peptides and corresponding precursor proteins, GO and pathway analyses were performed. The cellular components, molecular functions, biological processes, and putatively involved pathways of these peptides and precursor proteins were investigated by enrichment analysis. Regarding cellular components, organelle part, membrane part, intracellular organelle, cell part, and cell were the dominant enriched subcategories (Figure 4A). For molecular function, structural molecule activity and enzyme binding were the most highly enriched subcategories (Figure 4B). For biological processes, wound healing, response to wounding, response to stress, regulation of biological quality, and biological regulation were the most highly enriched subcategories (Figure 4C). Pathway analysis indicated that the main precursor proteins were participated in the regulation of the actin cytoskeleton, phagosome, gap junction, and focal adhesion (Figure 4D).

### Interaction Network Analysis

**Figure 5:**
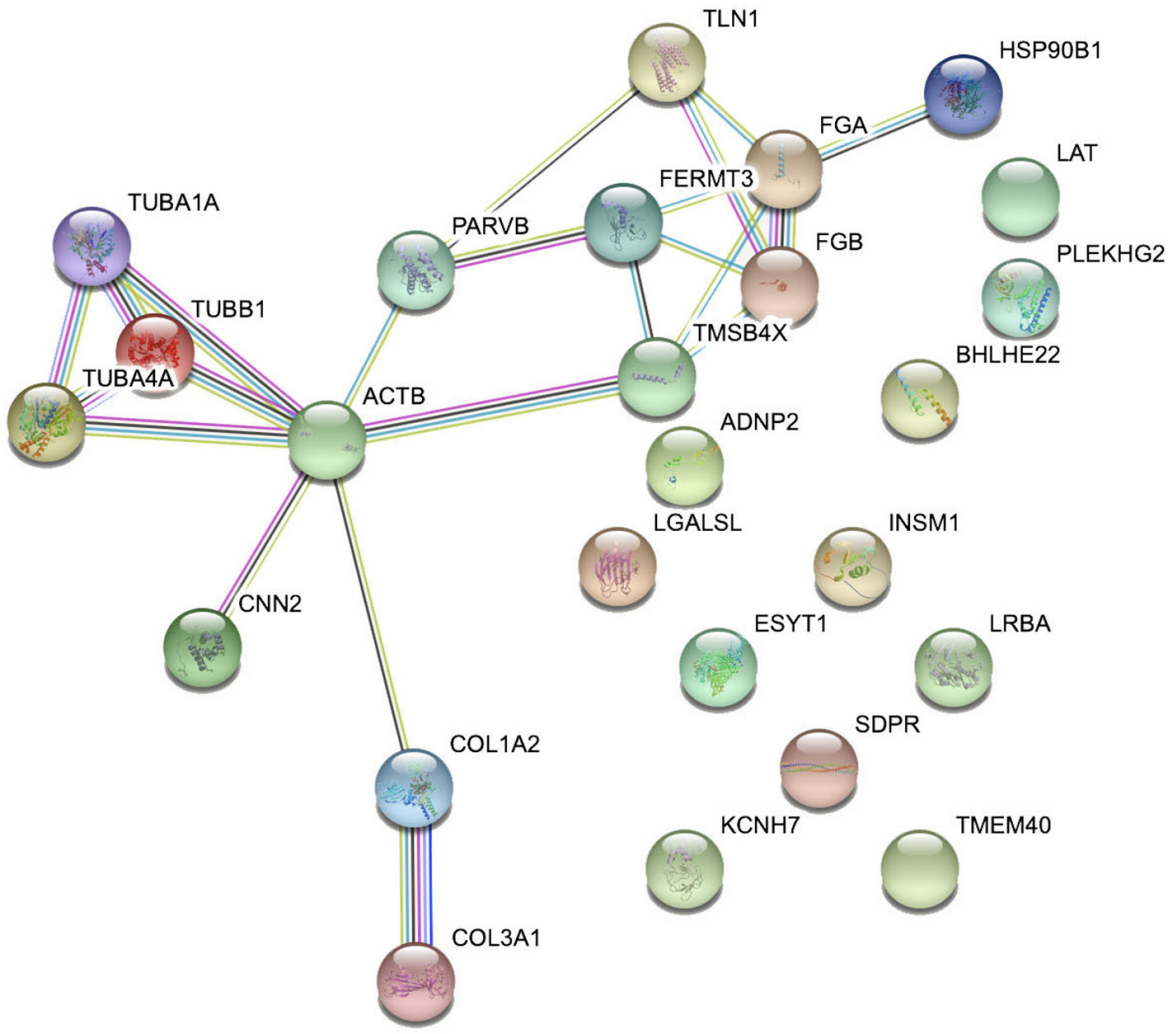
Interaction network analysis of precursor proteins of differentially expressed peptides according to STRING.

Interaction network analysis by STRING was performed to uncover potential interactions between differentially expressed peptides and relative precursor proteins. The results suggested that 14 were related to each other (Figure 5). Furthermore, we predicted the bioactivity of the differentially excreted peptides by PeptideRanker and found that there were 15 peptides with probabilities of bioactivity higher than 0.50 that came from five precursor proteins (Table 3). Among these five precursor proteins, fibrinogen alpha chain (FGA), collagen type 1 alpha 2 chain (COL1A2), collagen type III alpha 1 chain (COL3A1), and talin1 (TLN1) interact with each other (Figure 5).

**Table 3:** Predicted the bioactivity of the part differentially excreted peptides by peptideranker (bioactivity > 0.50).

**Peptide**	**Number**	**Foldchange**	**Predicted bioactivity**	**Protein name**
DSGEGDFLAEGGGVRGPRV	19	0.5726	0.71	FGA
DLVPGNF	7	4.9385	0.66	FGA
GEGDFLAEGGG	11	0.4916	0.64	FGA
NGEAGSAGPPGPPG	14	0.6826	0.62	COL1A2
QGLPGTGGPPGENGKPG	17	1.6492	0.62	COL3A1
SGEGDFLAEGGGV	13	0.5085	0.61	FGA
DSGEGDFLAEGGG	13	0.5693	0.55	FGA
DSGEGDFLAEGGGV	14	0.5209	0.55	FGA
EGDFLAEGGGVR	12	0.482	0.54	FGA
EGDFLAEGGG	10	0.5163	0.53	FGA
ADSGEGDFLAEGGGVR	16	0.8125	0.52	FGA
ACQSLGEPGCTQAQVLS	17	1.5496	0.52	TLN1
DAPGEVVPSGESGLR	15	3.3882	0.52	TMEM40
EGDFLAEGGGV	11	0.5169	0.51	FGA
VNDNEEGFF	9	1.6972	0.5	FGB

## Discussion

In the current study, we screened and identified serum peptide expression in neonatal umbilical cord blood and compared the different peptide profiles in the serum of umbilical cord blood of neonates with HIE and normal controls. This study was the first to identify potential endogenous functional peptides that may be involved in the progression of HIE and served as novel diagnostic biomarkers of HIE.

To investigate the pathogenesis of HIE, there are several reports on studying RNA (including miRNA, lncRNA, and circRNA) and protein profiling using blood samples from HIE patients (22–24). Zhu et al. (25) reported differential proteomic profiles in neonate blood with mild, moderate, severe, or without HIE and identified a total of 51 commonly differentially expressed proteins between HIE and healthy controls (25). Peptides are naturally generated in the body through protein degradation or restricted proteolysis (21). More than 13,000 natural peptides have been identified, and these usually play important roles in human physiology (26). Approximately 60 FDA-approved therapeutic peptides are on the market, and over 140 peptide-based drugs are currently in clinical development (27). It has been recognized that peptides play a vital role in the maintenance of central nervous system function. These include tridecapeptide neurotensin, cholecystokinin, neuropeptide Y, and amylin (28). In addition to their therapeutic effect, peptides can also be used as biomarkers for disease diagnosis (29). Here, we demonstrated that 49 peptides were differentially expressed in the serum of umbilical cord blood of the HIE group compared with the control group by performed LC-MS/MS. Thirty-three upregulated peptides and 16 downregulated peptides which were derived from 25 precursor proteins. These differential peptides suggest that they may be new biomarkers for the early diagnosis of HIE.

Most notably, 21 differentially expressed peptides were derived from the fibrinogen chain family in a total of 49 identified differentially expressed peptides. Among these peptides, 18 peptides were derived from the fibrinogen alpha chain (FGA), and three peptides were generated from the fibrinogen beta chain (FGB). Fibrinogen is an important blood coagulation protein and participates in numerous brain pathologies, subarachnoid hemorrhage, multiple sclerosis, and Alzheimer's disease (30–33). Fibrinogen inhibits oligodendrocyte progenitor cell (OPC) differentiation and myelination by activating the bone morphogenetic protein (BMP) signaling pathway (34). In addition, fibrinogen promotes cognitive deficits by CD11b/CD18-mediated microglial activation of Alzheimer's disease (AD) pathogenesis (35). Furthermore, fibrinogen is a potential biomarker for multiple sclerosis and Alzheimer's disease (36, 37). Neurons and astrocytes have been reported to have the ability to express FGA and FGB, which indicates that the FGA- and FGB-derived peptides may be involved in the HIE pathological processes and may have implications for the early diagnosis of HIE.

In conclusion, peptidomic analysis was performed by LC-MS and used to detect differentially expressed peptides between neonates with HIE and controls. The present study is the first to examine the potential relationship between peptides and HIE in newborns. In addition, GO and KEGG pathway analyses were used to identify the potential functions of the precursor proteins. This study provides a new view for further understanding the progress of HIE and may provide a new biomarker for the detection of HIE. However, the relevance of those peptides in umbilical cord blood to HIE need to be further investigated.

## Data Availability Statement

The original contributions presented in the study are included in the article/supplementary material, further inquiries can be directed to the corresponding author/s.

## Ethics Statement

The studies involving human participants were reviewed and approved by the Medical Ethics Committee of Jingjiang People's Hospital in China [approval no. (2020)38]. Written informed consent to participate in this study was provided by the participants' legal guardian/next of kin.

## Author Contributions

XD, LY, and YZha designed the project and edited the manuscript. YS and QS wrote the manuscript. JZ collected the clinical data and revised the manuscript. XW and YZhu participated in the design of this study. All authors contributed to the article and approved the submitted version.

## Conflict of Interest

The authors declare that the research was conducted in the absence of any commercial or financial relationships that could be construed as a potential conflict of interest.

## Publisher's Note

All claims expressed in this article are solely those of the authors and do not necessarily represent those of their affiliated organizations, or those of the publisher, the editors and the reviewers. Any product that may be evaluated in this article, or claim that may be made by its manufacturer, is not guaranteed or endorsed by the publisher.
